# From research to clinical practice: a systematic review of the implementation of psychological interventions for chronic headache in adults

**DOI:** 10.1186/s12913-020-05172-y

**Published:** 2020-05-25

**Authors:** Cinzia Perlini, Valeria Donisi, Lidia Del Piccolo

**Affiliations:** 1 Section of Clinical Psychology, Department of Neurosciences, Biomedicine and Movement Sciences, University of Verona, Verona, Italy; 2 USD Clinical Psychology, Azienda Ospedaliera Universitaria Integrata of Verona, Verona, Italy

**Keywords:** Chronic headache, Migraine, Tension-type headache, Psychological interventions, Behavioral interventions, Cognitive-behavioral therapy (CBT), Mindfulness, Acceptance and commitment therapy (ACT), Biofeedback (BFB), Relaxation training

## Abstract

**Background:**

Psychological interventions have been proved to be effective in chronic headache (CH) in adults. Nevertheless, no data exist about their actual implementation into standard clinical settings. We aimed at critically depicting the current application of psychological interventions for CH into standard care exploring barriers and facilitators to their implementation. Secondarily, main outcomes of the most recent psychological interventions for CH in adults have been summarized.

**Methods:**

We conducted a systematic review through PubMed and PsycINFO in the time range 2008–2018. A quality analysis according to the QATSDD tool and a narrative synthesis were performed. We integrated results by: contacting the corresponding author of each paper; exploring the website of the clinical centers cited in the papers.

**Results:**

Of the 938 identified studies, 28 papers were selected, whose quality largely varied with an average %QATSDD quality score of 64.88%. Interventions included CBT (42.85%), multi-disciplinary treatments (22.43%), relaxation training (17.86%), biofeedback (7.14%), or other interventions (10.72%). Treatments duration (1 day-9 months) and intensity varied, with a prevalence of individual-basis implementation. The majority of the studies focused on all primary headaches; 4 studies focused on medication-overuse headache. Most of the studies suggest interventions as effective, with the reduction in frequency of attacks as the most reported outcome (46.43%). Studies were distributed in different countries, with a prevalent and balanced distribution in USA and Europe. Ten researches (35.71%) were performed in academic contexts, 11 (39.28%) in clinical settings, 7 (25%) in pain/headache centres. Interventions providers were professionals with certified experience. Most of the studies were funded with private or public funding. Two contacted authors answered to our e-mail survey, with only one intervention implemented in the routine clinical practice. Only in three out of the 16 available websites a reference to the implementation into the clinical setting was reported.

**Conclusion:**

Analysis of contextual barriers/facilitators and cost-effectiveness should be included in future studies, and contents regarding dissemination/implementation of interventions should be incorporated in the professional training of clinical scientists. This can help in filling the gap between the existing published research and treatments actually offered to people with CH.

## Background

Chronic headache (CH) represents a clinical condition valued as one of the top 20 causes of disability worldwide [1, 2]. According to the third edition of the International Classification of Headache Disorders (ICHD-3 [3]), primary headache includes migraine, tension-type (TTH), cluster and mixed type headache, whose chronic form requires at least 15 days/month of pain for at least 3 months. Given the frequency and intensity of headache attacks, CH has a strong detrimental influence on patients’ quality of life with a high level of reported distress [4, 5]. Also, a greater percentage of psychopathology including depression and anxiety symptoms characterizes chronic migraineurs compared to non-chronic subjects or healthy controls [5, 6].

Traditionally, the main treatment of CH is represented by medication, with a frequent risk of overuse (Medication overuse Headache, MoH). However, medication alone shows moderate efficacy [7]. Headache and especially chronic migraine represent a multifaceted disease in which psychological factors play a crucial role both as triggers of headache attacks and maintenance factors [8]. In particular, locus of control, self-efficacy, social support, and emotional states have been described as influencing the course of the disease, by affecting perceived pain, migraine management, and the overall impact of headache on related disability and quality of life [9, 10]. In line with this bio-psychosocial model of chronic migraine, in the last years, multi-componential approaches have been suggested and developed which include pharmacological, psychological and/or physical components [11–15]. In particular, psychological interventions have been proposed either as complementary to pharmacological treatment or as a stand-alone therapy [16–18]. They have been generally grouped into three categories: a) relaxation training, b) biofeedback (BFB), and c) cognitive-behavioral therapy (CBT). CBT includes stress management training, and two main approaches of the third wave CBT therapy such as Acceptance and Commitment Therapy (ACT), and mindfulness [19, 20]. Such behavioral interventions are especially effective in reducing headache-related disability and affective distress in patients who do not respond to medication and in the prevention of attacks, with grade-A evidence of the effectiveness of biofeedback [21]. Overall, previous studies emphasize the role of psychological interventions in preventing headaches, reducing the frequency and severity of attacks, headache-related disability, and affective distress and providing patients with strategies to manage the physiological and psychological components of the disease [17–21]. Interestingly, the recent rise of information-technology-based approaches has contributed to the preliminary administration of behavioral therapies through different devices and online [22], although further researches are warranted to test the feasibility and the effectiveness of these new therapeutic approaches.

Compared to the big amount of published literature assessing the feasibility and effectiveness of behavioural interventions for adult people with CH in research settings, studies detailing the implementation of psychological approaches into the standard care of CH are quite rare (i.e. [23]). ‘Implementation’ refers to the use of strategies to adopt and integrate evidence-based health interventions and change practice patterns within specific settings’ [24]. Nowadays, a gap seems to exist between the knowledge on the effectiveness of behavioural interventions as described by scientific literature and their implementation into clinical contexts after the conclusion of the research and the publication of results [25–27]. Indeed, although effectiveness represents one of the aspects ensuring quality of care, a full assessment of quality of services also requires contemplation of access [28]. A comprehensive consideration of such factors is actually missing in the existing literature of psychological intervention for CH in adult population.

In order to reach a better understanding of these aspects, the main aims of the present paper are: 1) to assess the current implementation of psychological interventions described in the literature of CH into routine practice; 2) to analyse factors possibly representing barriers or facilitators of implementation (such as funding, setting, healthcare providers) and the possible gaps to the implementation (such as geographical context, target population, kind of implemented approaches). Finally, we briefly summarized the effective outcomes of the most recent psychological interventions for CH.

## Methods

**Fig. 1:**
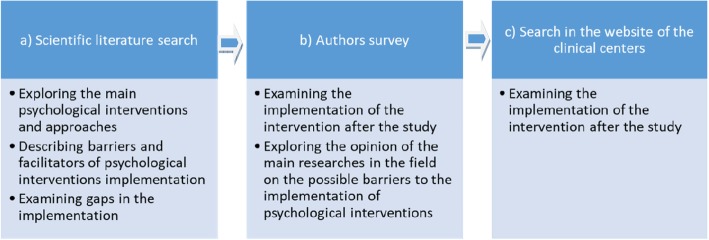
Multicomponent approach including three consecutive research methodologies

In order to reach our aims, we used a multidimensional approach consisting of three steps: a) searching relevant literature; b) contacting the corresponding author; c) visiting the website of the clinical centers cited in the selected papers (where available). Strategies b) and c) aimed at obtaining additional information with respect to those described in the literature (see Fig. 1).

### Searching relevant literature

The search and selection of relevant studies related to our topic were performed in accordance with the PRISMA (Preferred Reporting Items for Systematic reviews and Meta-Analyses) guidelines [29].

#### Search strategy

We identified studies focused on psychological interventions for chronic headache through searching the electronic databases PubMed and PsycINFO (research date: January 18, 2019). Three sets of keyword search algorithms were used linked with the Boolean operator ‘AND’. The first keyword included the terms “migraine” OR “headache. The second one included the term “chronic“. The third set of keyword search was related to the intervention and included the term “psychol* intervention”. The final search formula was: [((((migraine) OR headache)) AND chronic) AND psychol* intervention]. Filters for English language and publication date (1st January 2008-31st December 2018) were applied.

#### Study selection criteria

The following inclusion criteria were used: 1) adult population (age > 18 years); 2) diagnosis of chronic primary headache (migraine, TTH, mixed headache); 3) the study has to focus on the administration of psychological interventions for chronic primary headache (i.e. the term ‘chronic’ is specified in the title) with or without MoH, or severe headache (presence of MoH; without MoH but with high triptan or analgesic intake frequency; Migraine Disability Assessment-MIDAS score > 5; no maximum number of days or attacks specified); 4) psychological interventions which have shown to be evidence-based such as cognitive-behavioral therapy (CBT), mindfulness and acceptance and commitment therapy (ACT), biofeedback, relaxation training; 5) peer reviewed research articles; 6) observational studies, retrospective or prospective cohort studies, randomised trials, qualitative and mixed methods studies; 7) English language; 8) publication date between 1st January 2008 and 31 December 2018 (including e-pub). The publication time range was selected in relation to the objective of our work that aimed primarily at understanding which factors contribute to or impede the implementation of the most recent and evidence-based psychological interventions, by including also the researcher survey and the websites exploration. In our opinion ten years is an appropriate time frame to be able to contact researchers who are still active in this field and to have an appropriate evaluation of the factors related to clinical implementation in standard care of the most recent evidence-based treatments of CH.

Exclusion criteria were: 1) review papers, study protocols, conference abstracts or posters, books or book chapters and case reports; 2) papers not focusing on psychological interventions; 3) papers focusing on secondary analysis (i.e. referring to the same sample and intervention); 4) papers in which the maximum number of headache days were specified in the inclusion criteria was lower than expected for a diagnosis of CH (i.e. “Headache attack frequency had to be between one and six attacks per month”); 5) papers explicitly excluding chronic condition (i.e. “subjects with more than 14 headache days/month were not enrolled”); 6) studies on general chronic pain where it was not possible to distinguish the diagnosis of CH or studies on general chronic pain where it is possible to identify the sub-sample with CH but the percentage of participants with CH was less than 50% (in order to be sure that psychological interventions were focused on samples made up by the majority of the patients affected by CH).

#### Selection procedure

**Fig. 2:**
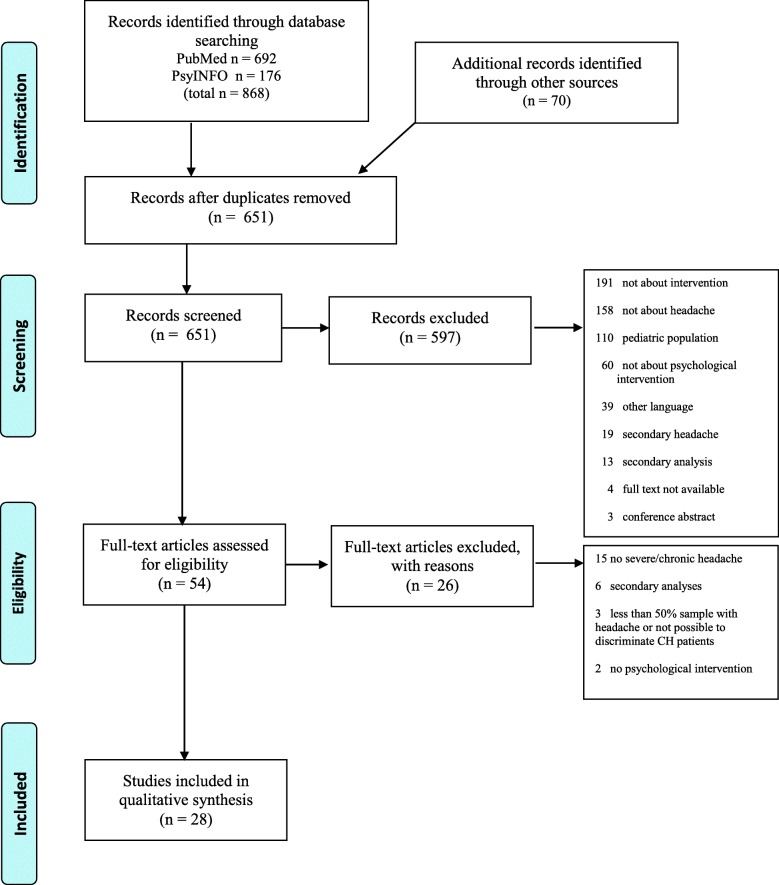
PRISMA flow diagram of the study selection

Both C.P. and V.D. interrogated independently the databases and screened title and abstract of the resulted items. Whenever at least one author raised concerns about study inclusion, the full text was inspected until a consensus was reached. When a consensus was not achieved, the third author (L.D.P.) checked the information and after further discussion a final decision was taken. For all search items that passed the first screening, we reviewed the full texts. Also, we cross-referenced lists of included studies to identify papers that the search terms had not found. Finally, since a notable number of reviews exist on behavioral intervention for primary CH, we retrieved and reviewed them and selected potential further papers published between 1st January 2008 and 31 December 2018 (see Fig. 2).

#### Data collection process and data items collected

For each included paper the first author (C.P.) extracted the main research variables using a standardized data extraction form (Microsoft Excel 2010). In particular, we focused on aspects of the study potentially representing source of information on barriers or factors helping the implementation of interventions into standard care. Extracted variables were: first author, year of publication, setting and country, hospital/clinical centre involved in recruitment and/or intervention (if mentioned in the study), website of the hospital/clinical centre, pathology, number of participants included in the study and number of those analysed, recruitment, study design, type of psychological intervention, administration (individual and/or group and additional delivery mode), intervention provider, length of intervention, percentage of adherence to intervention, main results, funding, implementation into clinical context at the end of research [as specified in the paper and/or by visiting website, see c)].

#### Risk of bias assessment in individual studies

All eligible studies were evaluated against the 16-item quality assessment tool (QATSDD) [30]. The tool shows good reliability and validity for use in the quality assessment of methodologically diverse set of research articles. It consists of 16 criteria each with a score ranging between 0 (‘no mention at all’) and 3 (e.g. ‘detailed description of each stage of the data collection procedure’). The maximum possible score is 42 for qualitative or quantitative studies and 48 for mixed-method studies. For each included study, we added the item scores and divided the result by the maximum possible score (%QATSDD total score) to report the paper’s overall quality score. Furthermore, mean and standard deviation were calculated for each item in order to describe the items with higher and lower values [31–33]. Two authors (C.P. and V.D.) applied the QATSDD to included studies, and disagreements were resolved through discussion with the third author (L.D.P.) until consensus was achieved.

### Contacting the corresponding author by e-mail

The corresponding author of each included paper has been contacted by e-mail by asking him/her whether the intervention described in the published paper has been introduced in routine clinical settings. A 5-min survey (available as Supplementary material) has been created, regarding actual implementation, barriers or factors favoring successful application of the intervention. We specified in the e-mail that the collected data would have been used only for the present research and that no personal data were collected. We sent the initial e-mail on June 22, 2019, and subsequent 3 reminders (the first after 1 week from the first mail, the second after further 2 weeks and the third after one further week).

### Exploring websites

As an adjunctive strategy, we tried to obtain information about the implementation of interventions by visiting the clinical center website as specified in the paper full text. Only websites having an Italian or English version were consulted.

## Results

### Searching relevant literature - results

We retrieved 868 studies from electronic database and 70 from reviews and meta-analyses [17, 18, 20–22, 34–47]. After removing duplicates and applying exclusion criteria, we obtained a final selection of 28 papers [11–15, 23, 48–69] (see flow diagram of studies selection in Fig. 2). The most of the studies (17 out of 28, 60.71%) were randomized controlled trials (RCT) with a direct comparison between groups assigned to psychological interventions for CH versus waiting list or other approaches. Five RCTs were defined by authors as ‘pilot RCT’ because of the small sample size. The remaining papers were observational or retrospective studies. The following paragraphs summarize the variables of interest of the selected papers (see also Table 1 and Supplementary Table).

**Table 1:** Variables of the 28 selected studies (in chronological order)

Paper	Country	Setting	Pathology declared in the paper	Study design	Intervention	Intervention provider
D’Souza et al. 2008 [52]	USA	University	migraine and/or TTH	RCT	RT vs WED vs control	audiotape-guided exercises
Matchar et al. 2008 [48]	USA	University+ primary care setting	migraine and/or TTH or other primary headache	RCT	educational session (including information on headache type, pharmacologic treatment, triggers, sleep hygiene, and relaxation techniques) + diagnosis and treatment by a professional especially trained in headache care + proactive follow-up by a case manager vs usual care	mid-level provider (eg, nurse practitioner or PA) with expertise in headache evaluation and management
Sauro et al. 2008 [11]	CANADA	University	primary headache (MoH included)	observational	CHAMP Program includes: 1. Education Session2. Lifestyle Assessment3. Self-Management Workshop4. Nursing Contact and Advice5. Physician Visit	psychologist, nurse, physician, kinesiologist, occupational therapist, neurologist
Grazzi et al. 2009 [67]	ITALY	IRCCS (Institute for Treatment and Research)	migraine (after withdrawal from MoH)	observational, non-randomized	pharmacological treatment+limited-contact RT vs pharmacological treatment alone	ns
Gunreben-Stempfle et al. 2009 [12]	GERMANY	Tertiary pain center (University)	migraine and/or TTH and other primary headache (MoH included but discontinuation of medication in the case of MoH)	prospective cohort	high intensity 96-h multidisciplinary headache treatment program (CBT-SMT + PMR + physical exercices+education) vs low intensity 20-h program and primary care	qualified psychologist and neurologist
Holroyd et al. 2009 [60]	USA	University	TTH (MoH excluded)	RCT	AM vs PL vs CBT-SMT + PL vs CBT-SMT + AM	psychologist or masters level counselor
Fritsche et al. 2010 [53]	GERMANY	Headache center (University hospital)	migraine (prevention of MoH)	RCT	cognitive-behavioral MCP vs brochure (bibliotherapy) for the prevention of MoH	psychological psychotherapists with special professional education in pain therapy and long-standing experience in the treatment and research of headache syndromes, especially of drug-induced-headache
Gaul et al. 2011 [13]	GERMANY	Tertiary headache center (hospital)	migraine and/or TTH and/or MoH	prospective observational	MTP (individual CBT sessions, PMR, physical therapy, aerobe ergometer training, face-to-face appointment with neurologist and psychologist)	behavioral psychologist, physioterapist
Hedborg and Muhr 2011 [14]	SWEDEN	University	migraine and/or TTH	RCT	hand massage+multimodal behavioral treatment vs multimodal behavioral treatment vs control group.	online administration (program developed by authors together with a professional advisor in stress management)
Abdoli et al. 2012 [54]	IRAN	University hospital	TTH	RCT	GI (with tape or perceived happy memory) vs control group	certified and experienced psychoterapist
Bembalgi et al. 2012 [65]	INDIA	University hospital	TTH (MoH excluded)	RCT	auditory BF vs visual BF vs combined BF vs control group (only medication prescribed by their physician)	ns
Ezra et al. 2012 [23]	ISRAEL	University hospital	TTH (MoH included)	retrospective	HR vs amitriptyline	neurologist with hypnosis training
Mo’tamedi et al. 2012 [61]	IRAN	University+headache centre (hospital)	migraine and/or TTH	RCT	ACT+TAU vs TAU	experienced graduate hospital staff certified psychologist
Ruehlman et al. 2012 [50]	USA	Pain centre	migraine and/or TTH and/or cluster headache and other chronic pain conditions	RCT	CPMP vs TAU	online adiministration (content developed by a team of psychologists, employee assistance professional and physical therapist)
Wallasch et al. 2012 [15]	GERMANY-SWITZERLAND	Tertiary headache center (hospital)	migraine and/or TTH and other primary headache and/or MoH	prospective observational, non-randomized	3 modules (moderate, severe and severe with additional problem chronicity):1. education and patient self-management; 2. module 1 treatment and a MTP consisting of individual and group sessions of CBT, PMR etc.; 3. module 1 and 2 plus hospitalization	psychologist, neurologist, psysical therapist
Slavin-Spenny et al. 2013 [69]	USA	University	headache	RT	AAET vs RT vs waiting list	four female doctoral students in clinical psychology who were trained in interventions
Cathcart et al. 2014 [55]	AUSTRALIA	University	TTH (MoH excluded)	RCT pilot	brief MBT vs waiting list	experienced psychologist with formal mindfulness training
Day et al. 2014 [63]	USA	University+ headache centre	migraine and/or TTH or other primary headache (MoH included)	RCT pilot	MBCT vs DT	advanced graduate student in clinical psychology and licensed clinical psychologist with extensiveexperience in the treatment of pain. A certified yoga instructor conducted the guided mindful movement segment
Martin et al. 2014 [66]	AUSTRALIA	University	migraine and/or TTH (MoH excluded)	RCT	LCT (including desensitization) vs avoidance vs CBT + avoidance vs waiting list	two doctoral-trained psychologists
Christiansen et al. 2015 [56]	GERMANY	University hospital	migraine and/or TTH (MoH excluded)	single-group outcome study	CBT (psychoeducation, PMR, coping strategies for pain and stress, and goal setting skills)	a team of five clinical psychologists
Cousins et al. 2015 [68]	UK	University	migraine (MoH included)	RCT pilot	brief guided self-help CBT and relaxation vs standard medical care	trained CBT therapist supervised by senior CBT therapist
Bakhshani et al. 2016 [64]	IRAN	University+ hospital	migraine and/or TTH	RCT	MBSR+drug vs drug	ns
Rausa et al. 2016 [51]	ITALY	University hospital+clinical centre	migraine and/or TTH (focused on MoH)	RCT pilot	frontal BFB + prophylactic pharmacological therapy vs prophylactic pharmacological therapy+weekly sessions with a psychologist (interviews about previousweek’s headaches, mood, and analgesic intake)	ns for BFB; psychologist for control group
Smitherman et al. 2016 [57]	CANADA	University hospital	migraine with comorbid insomnia (MoH excluded)	RCT pilot	CBT for insomnia vs control group	three graduate-level therapists with backgrounds in cognitive-behavioral therapy and behavioral medicine
Grazzi et al. 2017 [59]	ITALY	IRCCS (Institute for Treatment and Research)	migraine (after withdrawal from MoH)	exploratory	MBSR vs medication	experienced neurologist trained in mindfulness practice
Krause et al. 2017 [49]	USA	Hospital	primary and secondary headaches (MoH included)	prospective cohort	BFB, psychotherapy, psycho-educational group, group family meeting+nursing +medical+physical modules	ns
Wachholtz et al. 2017 [58]	USA	University	migraine, mixed migraine	RCT	spiritual meditation vs internally focused secular meditation vs externally focused meditation vs PMR	research assistants who were trained on the study protocols
Minen et al. 2019 [62]	USA	University hospital	migraine	observational	RELAXaHEAD (app)	app

*AET* Anger Awareness and Expression Training, *ACT* Acceptance and Commitment Therapy, *AM* tricyclic antidepressant medication, *BFB* biofeedback, *CBT* Cognitive Behavioral Therapy, *CHAMP* Calgary Headache Assessment And Management Program, *CPMP* Chronic Pain Management Program, *DT* delayed treatment, *GI* guided imagery, *LCT* Learning to Cope with Triggers, *HR* hypnotic relaxation, *MBCT* Mindfulness-Based Cognitive Therapy, *MBSR* Mindfulness-Based Stress Reduction training, *MBT* mindfulness-based training, *MCP* Minimal Contact Program, *MoH* Medication overuse Headache, *MTP* Multidisciplinary Treatment Program, *ns* not specified, *PMR* Progressive Muscle Relaxation, *PL* placebo, *RCT* Randomized Controlled Trial, *RT* Relaxation Training, *SMT* Stress-Management Therapy, *TAU* Treatment As Usual, *TTH* Tension Type Headache, *WED* Written Emotional Disclosure

#### Intervention

Twelve studies (42.85%) used CBT approaches (mindfulness-based training, ‘learning to cope with triggers’ program, ACT, guided imagery), 6 (22.43%) described multi-disciplinary interventions including at least one psychological component, 5 (17.86%) were based on relaxation training, 3 (10.72%) compared different psychological interventions, and 2 focused on BFB (7.14%). Among them, 12 (42.85%) had an individual-based approach, 9 (32.14%) were group-based interventions, and 7 (25%) studies combined individual plus group activities. In 12 studies (42.85%) the intervention included additional delivery mode as homework and e-mail/telephone support. Interventions covered the duration range of 1 day-9 months. Adherence to treatment ranged from to 37.5 to 100%.

#### Pathology declared in the paper

The most of the studies (17 out of 28; 60.71%) included patients with all diagnosis of primary headache (migraine, TTH, mixed or cluster headache), 5 (17.85%) were focused only on migraine and 5 only on TTH. One study [49] included both primary and secondary headaches. Among them, twelve studies included patients with MoH (42.85%), 6 (21.43%) studies excluded these patients, and 10 (35.71%) studies did not specify the inclusion of MoH either in inclusion/exclusion criteria or in sample characterization. Among the 12 studies including patients with MoH, 4 were specifically focused on the prevention or treatment of MoH (i.e. during or after withdrawing).

#### Effectiveness of interventions

Most of the studies suggest psychological interventions as effective in improving at least one headache outcome. In particular, a reduction in frequency of attacks is the most reported finding (13 studies: [12–15, 51–59]), followed by reduction in pain-related disability (7 studies: [11, 15, 49, 50, 52, 60, 61]), symptoms of anxiety, depression, stress (7 studies: [12, 15, 48–50, 61, 62]), sensory perception of pain (i.e. intensity, duration; 7 studies: [23, 50, 52, 54, 56, 63, 64]), use of medication [15, 53, 59, 65], catastrophizing [50, 56, 63]. Improvements were observed in quality of life [48, 64–66], coping strategies [51, 56], mindfulness ability [55], self-efficacy [63]. In over 80% of the studies comparing psychological intervention with treatment as usual (TAU) or waiting list, behavioral approaches overtook the control group in at least one headache outcome.

#### Country

Among the selected studies, 10 out of 28 (35.71%) were carried out in Europe (Germany, Italy, Sweden, Switzerland, United Kingdom), 11 (39.28%) in North-America (Canada, USA) and 7 (25%) in other countries (Australia, India, Iran, Israel).

#### Setting

Ten researches (35.71%) were performed in academic contexts (University), with participants usually recruited among the general population or undergraduate/college/university students (usually using advertisement and media) or referred by local physicians/neurologists. Among them, one study [48] was carried out with the collaboration between University and three different primary care settings. Of the remaining studies, 11 (39.28%) were carried out in clinical settings with participants enrolled among patients referring to i.e. the departments of neurology and/or anaesthesiology of general (University) hospitals; 7 (25%) in specialized pain/headache centres (2 of them were private and the remaining 5 were part of hospitals or universities. Among the latter, 3 were classified as tertiary headache centers, all situated in Germany).

#### Intervention provider

Health providers who administered interventions were certified psychologists or psychotherapist with extensive experience in the administered treatment and/or in the management of chronic pain conditions (9 studies, 32.14%); research assistants, students in clinical psychology or doctoral trained psychologist (3 studies, 10.72%); trained neurologists (2 studies, 7.14%); a multi-disciplinary team including (depending on the study) at least two among psychologists, neurologists, physical therapist, physiotherapists, occupational therapist, nurses (4 studies, 14.28%); mid-level providers (i.e. nurse practitioner or physician assistant) with expertise in headache evaluation and management (1 study, 3.57%). In 4 studies (14.28%) interventions were delivered online or by smartphone application (app) or employing audiotape. In two of these cases, it was specified that the content of the intervention was developed by the team of authors together with a professional advisor in stress management [14] or a team of psychologists, employee assistance professional and physical therapist [50]. In [51] a psychologist delivered the intervention to the control group while it was not specified who provided the experimental intervention (biofeedback). Finally, 4 studies (14.28%) did not specify the intervention provider.

#### Funding

Heighteen out of 28 studies (64.29%) were acknowledged as funded. In particular, they were granted by private (2 studies), public (6 studies) or both private and public (7 studies) funding (1 did not specify the nature of grant [63]). In one case [54] it was specified that the certified psychologist who administered the intervention was part of the clinical staff. Other three studies specified that all costs were covered by the patients’ health insurance [12], or that funding covered all costs with the exception of clinical care, so that patients’ insurers were billed for clinic visits [48] or specified solely that clinical costs were paid by health insurances [13]. Three (10.71%) studies declared no funding. Finally, 5 (17.86%) studies did not specify any funding in the acknowledgments section or elsewhere but in two cases it is possible to infer from the full text that personnel who delivered the intervention was part of the hospital tenured staff [15, 61].

To sum up, different kind of interventions were applied, mainly using an individual basis and a face to face implementation, with a prevalence of CBT approaches and treatment duration ranging from 1-day to 9 months, having them a different degree of intensity. Such interventions targeted patients mainly with primary CH, with only few explicitly excluding patients with MoH and 4 focusing on them. As regarding the effectiveness of these interventions, a reduction in frequency of attacks was the most reported outcome, observed in 13 out of 28 studies. Retrieved studies were distributed in different countries, with a prevalent and balanced distribution in USA and Europe. The setting of the interventions included hospitals, university hospitals, pain centers, (tertiary), headache centers (affiliating to university or hospital), Institutes for Treatment and Research-IRCCS, primary care settings. Intervention providers in most cases had high qualification or were multidisciplinary teams. Most of the studies were funded with private or public funding.

#### Evaluation of the methodological quality of selected papers

Overall, the quality of the included studies was variable; the %QATSDD score ranged between 38.10% (mean raw score = 16) [67] and 80.95% (mean raw score = 34) [14], with an average quality score for all papers of 64.88% (raw score of 27.25). Only two studies resulted under the 50% of the total score (raw score of 21) (Table 2). Variations in quality among studies concerns the description of the research setting, the explanation of the rationale for the choice of data collection tools and the presence of statistical assessment of reliability and validity of tools themselves. The lowest QATSDD single item score was referred to user involvement in the design of the study, with only six studies reporting it (item mean score ± SD = 0.39 ± 0.83) [11, 13, 14, 23, 50, 62]. Also, a representative sample of target group of reasonable size is absent in most studies due to recruitment among general population/students or small patients sample size for each type of headache diagnosis (item mean score ± SD = 1.25 ± 0.44) [11–15, 23, 49–51, 55–58, 60, 62–64, 66–68]. Sample size considered in terms of analysis is lacking in 18 out of 28 studies (item mean score ± SD = 1.04 ± 1.43) [11–13, 15, 23, 49, 50, 52, 54, 55, 57, 59–61, 63, 64, 67]. Finally, most papers discussed limitations in details, but only three critically mentioned the strengths of the research (item mean score ± SD = 1.86 ± 0.59) [50, 53, 56].

**Table 2:** Assessment of studies’ quality based on QATSDD method

Study	Explicit theoretical framework	Statement of aims/objectives in main body of report	Clear description of research setting	Evidence of sample size considered in terms of analysis	Rapresentative sample of target group of a resonable size	Description of procedure for data collection	Rationale for choice of data collection tool(s)	Detailed recruitment data	Statistical assessment of reliability and validity of measurement tool(s)*	Fit between stated research question and method of data collection*	Fit between stated research question and method of analysis	Good justification for analythical method selected	Evidence of user involvement in design	Strenghts and limitations critically discussed	QATSDD Total Score	% QATSDD Total Score
D’Souza et al. 2008 [52]	3	3	1	0	1	3	1	3	0	3	2	0	0	2	22	52.38%
Matchar et al. 2008 [48]	3	3	3	3	2	3	1	3	0	3	2	2	0	2	30	71.43%
Sauro et al. 2008 [11]	2	3	3	0	1	3	2	3	0	2	2	1	1	2	25	59.52%
Grazzi et al. 2009 [67]	1	3	2	0	2	2	1	2	0	2	0	0	0	1	16	38.10%
Gunreben-Stempfle et al. 2009 [12]	2	2	3	0	1	3	2	3	1	2	2	3	0	2	26	61.90%
Holroyd et al. 2009 [60]	2	1	1	0	1	3	2	2	1	3	2	3	0	1	22	52.38%
Fritsche et al. 2010 [53]	2	2	3	3	2	3	2	3	1	3	2	2	0	3	31	73.81%
Gaul et al. 2011 [13]	2	2	3	0	1	3	0	3	0	2	2	2	1	2	23	54.76%
Hedborg and Muhr 2011 [14]	2	3	3	3	1	3	2	3	2	3	2	3	2	2	34	80.95%
Abdoli et al. 2012 [54]	3	3	2	0	2	3	0	2	0	3	2	3	0	1	24	57.14%
Bembalgi et al. 2012 [65]	2	3	2	3	2	3	3	2	1	3	2	3	0	2	31	73.81%
Ezra et al. 2012 [23]	2	2	3	0	1	2	0	2	0	2	2	0	2	1	19	45.24%
Mo’tamedi et al. 2012 [61]	3	3	3	0	2	3	3	2	3	3	2	3	0	2	32	76.19%
Ruehlman et al. 2012 [50]	3	3	2	0	1	3	1	3	1	3	2	3	2	3	30	71.43%
Wallasch et al. 2012 [15]	2	2	3	0	1	3	2	2	1	2	2	2	0	1	23	54.76%
Study	Explicit theoretical framework	Statement of aims/objectives in main body of report	Clear description of research setting	Evidence of sample size considered in terms of analysis	Rapresentative sample of target group of a resonable size	Description of procedure for data collection	Rationale for choice of data collection tool(s)	Detailed recruitment data	Statistical assessment of reliability and validity of measurement tool(s)*	Fit between stated research question and method of data collection*	Fit between stated research question and method of analysis	Good justification for analythical method selected	Evidence of user involvement in design	Strenghts and limitations critically discussed	QATSDD Total Score	% QATSDD Total Score
Slavin-Spenny et al. 2013 [69]	3	3	2	3	1	3	2	3	3	3	2	2	0	2	32	76.19%
Cathcart et al. 2014 [55]	3	2	2	0	1	3	2	3	3	3	2	2	0	1	27	64.29%
Day et al. 2014 [63]	3	3	3	0	1	3	3	3	3	3	2	2	0	2	31	73.81%
Martin et al. 2014 [66]	3	2	1	2	1	3	2	2	3	2	2	2	0	2	27	64.29%
Christiansen et al. 2015 [56]	3	3	3	3	1	3	2	3	3	2	2	2	0	3	33	78.57%
Cousins et al. 2015 [68]	3	3	2	3	1	3	2	3	1	3	2	2	0	2	30	71.43%
Bakhshani et al. 2016 [64]	3	3	3	0	1	3	2	3	3	3	2	2	0	1	29	69.05%
Rausa et al. 2016 [51]	3	3	3	3	1	3	2	3	3	3	2	2	0	2	33	78.57%
Smitherman et al. 2016 [57]	3	3	1	0	1	3	2	3	3	3	2	2	0	2	28	66.67%
Grazzi et al. 2017 [59]	3	3	3	0	2	3	2	3	2	2	2	2	0	2	29	69.05%
Krause et al. 2017 [49]	1	3	3	0	1	3	0	3	0	2	2	2	0	2	22	52.38%
Wachholtz et al. 2017 [58]	3	3	3	3	1	3	1	1	3	3	2	2	0	2	30	71.43%
Minen et al. 2019 [62]	2	3	2	0	1	1	1	1	3	2	2	1	3	2	24	57.14%
MEAN	2.50	2.68	2.43	1.04	1.25	2.86	1.61	2.57	1.57	2.61	1.93	1.96	0.39	1.86	27.25	
Standard Deviation	0.64	0.55	0.74	1.43	0.44	0.45	0.88	0.63	1.29	0.50	0.38	0.88	0.83	0.59	4.62	

Score: 0 = Not at all; 1 = Very slightly; 2 = Moderately; 3 = Complete

Two items: 1) ‘Fit between stated research question and format and content of data collection tool’; 2) ‘Assessment of reliability of analytical process’ where not included, as they apply only to qualitative studies

### Contacting authors by e-mail - results

Of the 28 authors contacted, 2 (7.14%) replied to our e-mail filling in the survey. In one case the author specified that the intervention described in the related paper is nowadays routinely applied but with some adjustments. He/she also pointed at the lack of qualified staff and missing support by the management as main obstacle factors to the implementation of the intervention into clinical practice (choosing among multiple choice answers; see our survey in the Supplementary material). Finally, he/she underlined the availability of a multidisciplinary team (together with the availability of a Clinical Psychological Service or qualified psychological staff) as a facilitating factor for the application of the intervention into routine context (choosing among multiple choice answers). In the other case, the author replied that the intervention was not routinely implemented after the end of the research because of a lack of funding (response selected by choosing among the multiple choice survey we sent).

### Visiting websites - results

The involvement of a clinical center as a site of recruitment and/or treatment was described in the full text of 18 out of 28 studies (64.29%). Among them, a webpage/website was available in 16 out of 18 cases (88.8%). Two studies shared the same website (hence only 1 website will be counted from here onward). Five websites were in English, 5 contained an English version, 5 did not have an English version (but 2 were in Italian). Twelve websites were hence visited searching for specific sections/pages describing psychological interventions offered for headache/migraine by the center (see Supplementary Table). In particular, 5 sites did not contain any information about psychological approaches to headache; two sites described psychological approaches offered to patients with headache, but treatments investigated in the published papers were not mentioned [51, 62]; one site (the one corresponding to 2 studies) contained a specific section focusing on treatment for chronic migraine but on the date of consultation (September 27, 2019) the page was ‘under construction’. In one case [49] it was not clear whether the described treatments were at least partly offered by the clinic. Only in three cases, in the corresponding website, there was a description of the treatment on which the studies were focused on, whit clear reference to the implementation of the treatment itself into the routine clinical setting [11, 23, 50].

## Discussion

The current paper investigated the implementation of the most recent psychological interventions for CH into routinely clinical settings by using three integrated methodologies and briefly summarizing the effectiveness of such interventions.

Different psychological approaches have been used in retrieved papers, covering all the three categories considered (RT, BFB and CBT). In particular, the majority of studies targeting adult patients with CH used CBT interventions, although the proposed approaches were very different in terms of aims, contents and structures, including standard CBT interventions such as coping with triggers and more recent approaches as mindfulness and ACT. Whereas mindfulness focus on directing attention to bodily sensations such as breathing, as well as non-judgmental awareness of the present [70], ACT is characterized by willingness to experience rather than control of pain and the pursuit of broader life values [71]. Such interventions appeared to be feasible, well-tolerated (medium-high percentage of adherence to treatments) and able to effectively impact at least one headache outcome, which is in line with previous reviews [18, 38, 42, 45]. In particular, we are nowadays assisting to gaining popularity of mindfulness and ACT because of their efficacy in the reduction of headache intensity [18] and in chronic pain treatment in general [72].

Around half of the retrieved studies included MoH patients, with one study specifically focusing on the prevention and three on the management of the withdrawal from MoH. This is of interest because the presence of MoH is particularly difficult to manage and patients suffering from this condition require multidisciplinary approaches including pharmacological and non-pharmacological therapies [59]. Despite that, the criteria of MoH has been not considered among the exclusion criteria in many studies. As for the proposed treatment, studies including MoH used multidisciplinary intervention (i.e. [11, 13, 15]) or compared the effectiveness of psychological intervention versus pharmacotherapy (i.e. [23, 51, 59, 67, 68]). Overall, they showed the favorable impact of multidimensional approaches on several CH dimensions in MoH such as a reduction of number of headache days/month [12, 13, 15], depressive symptoms [12, 15], headache-related disability [11, 15], and amount and intake frequency of medication [15]. Studies contrasting psychological and pharmacological approaches suggested at least equal (i.e. [59]) or superior (i.e. [51]) efficacy of psychological approaches especially in terms of compliance [23, 67] but also in symptoms relief [51, 67]. Such findings encourage future researches on the application of behavioural interventions in MoH.

Great heterogeneity emerged in terms of geographical context, indicating a diffuse relevance and need of therapies for CH, which is in line with recent recommendations of different headache agencies worldwide (i.e. USA: [73–75]; CANADA: [76]; BRASIL: [77]; UNITED KINGDOM: [78]; ITALY: [7]; FRANCE: [79]; SPAIN: [80]; GERMANY: [81]; JAPAN: [82]). Overall, they recommend that patients with headaches should undergo psychological therapy as an alternative or supplement to pharmacological treatment. In the same way, the Italian Consensus Conference on Pain in Neurorehabilitation (ICCPN) recommended electromyographic, thermal and electro galvanic biofeedback interventions (grade of recommendation A) in addition to autogenic training, relaxation training (grade B), hypnosis (grade C), and biofeedback intervention combined with virtual reality for the treatment of chronic TTH and migraine [21].

An interesting issue regarding possible barriers and facilitators to the implementation of interventions, is represented by funding. The presence of funding was acknowledged in more than half of the articles. Studies were supported by both private and public grants (or a combination of them) which usually were given for a limited time (months or few years) for research purposes, but not to plan clinical activities and implementation of the intervention in clinical routine. Financial costs are generally recognized as a pressing issue in the field of research [83] and a significant barrier deeply affecting the translation from research to clinical practice of empirically supported psychological interventions [84]. As an example, it has been observed that different payment methods (i.e. pay for performance, P4P), used by policymakers to transfer funds to health care providers (both individual or group professionals) can impact in different ways on the utilization of care facilities, especially by outpatients with chronic conditions [85]. In the case of the present work, one of the authors who filled in our survey indicated the impossibility of retrieving funding as the main reason for not continuing the administration of multi-componential intervention after the end of the research. In some cases, psychological interventions were covered by patients’ insurance coverage, which may vary depending on the specific insurance and on the health care system of the country. Patient willingness to pay for care in the headache clinic was considered by some authors as a criterion for the success of the intervention (i.e. [48]).

Moreover, it has to be said that different settings are not equivalent in terms of attracting devoted funding, recruiting high specialized professionals as part of the tenured staff, individualizing interventions, having a multidisciplinary team with ad hoc competences on the management of headache. If we hypothetically put them on a continuum, studies performed in purely academic settings and recruiting people among the general population are the farthest from the implementation of interventions (unless collaboration with a clinical center is established), while researches in tertiary headache/pain centers are the closest to. University hospitals or general pain centers which may benefit from some but not all advantages of more specialized centers stand in the middle of this continuum. Among the studies we selected and explored in detail, the majority of them (75%) were performed in purely academic settings or combined university-hospital contexts.

In over half of cases providers were healthcare professionals with high specialization, ad-hoc training, and expertise in the administration of the proposed intervention and/or in the management of chronic pain. Although it is not custom to detail in scientific papers people paid with a research grant, it is reasonable to infer that funded studies covered professionals’ salaries at least for the time of research. This is surely the case of [68] where it was clearly stated that the therapist was funded by a Charity, and of [11] in which it was specified that initial funding from the Alberta Medical Services Delivery Innovation Fund covered team salaries for three years, after which long term funding was provided to the program by a Regional Pain Program. Only in three cases, it was specified that the healthcare providers were part of the hospital/center clinical staff [15, 54, 61]. Interventions requiring high specialized professionals need more resources in terms of both salary and professional training, although it is likely that different amounts of training will be needed for different interventions. Of note, a recent study by Crome and colleagues [84] tested a hypothetical model taking into account potential professional training costs in relation with the adoption of a novel therapy, concluding that training costs should be carefully considered in decisions about implementing a psychological intervention.

The need of high-specialized professionals does not maximize cost-efficiency, especially when interventions were administered on individual-basis, representing a barrier to the continuation of interventions. However, it as to be noted that one out of three studies used group-based administration. A group-based delivery can be more effective than individual delivery [18] possibly because group-modality builds confidence, increases social interaction and promotes integration [86]. Furthermore, group-based approaches allow reducing the costs and improve the availability of the interventions [87, 88], similarly to e-health approaches, which have been introduced in 4 of the included studies. In the last years there is indeed a growing interest in the use of electronic behavioral interventions as well as mobile technologies such as smartphones for improving the care of CH. Nevertheless, the use of mobile devices is still scarce and further studies are warranted to establish the effectiveness and the degree of compliance of such new administration modalities. Also, the matter of security and privacy of health data should be addressed [22, 43].

To sum up, the implementation of psychological treatments for CH is possibly hampered by a combination of factors which should be taken into account by researchers and clinicians who will deal with the treatment of CH. For example, missing funding is reasonably related to people turnover, professionals training and to the difficulty in implementing high specialized interventions.

Information summarized in this paper give to researchers, clinicians and health agencies useful data on psychological approaches, healthcare professionals and targeted patients in the field of CH treatment. Also, the present work provides data on the geographical context and setting of the implementation of psychological interventions. Exploring these aspects highlighted possible gaps, barriers and facilitators to the implementation. However, this exploration did not allow to give a conclusive answer to our first question: are these psychological interventions available for patients in the routine clinical care? It has to be recognized that a great difficulty in retrieving information about what happened after the conclusion of researches emerged. Indeed, although all papers were interested in testing behavioral approaches aiming at ameliorating the clinical management of CH, few studies made a clear reference to the intent or possibility to implement the intervention into the local clinical context. Among them, [68] specified that it was a pilot study to provide design information necessary for a future definitive trial of the treatment within a United Kingdom-National Health System context. Also, [11] tested a multi-componential program that was developed in a public setting and concluded that ‘*with appropriate support from funding agencies, a multidisciplinary headache program can be successfully established as part of the Canadian public healthcare system*’. Furthermore, with few exceptions, the section of papers referring to the limitation of the study was almost always referred to research aspects like small sample size, missing of equivalent control group, etc. with no reference to clinical application.

In order to explore the availability of interventions, we also contacted the researchers. Unfortunately, this strategy did not allow to obtain any further useful information on the implementation of the interventions, with only two respondents. However, the utilization of this strategy, allowed to observe a turnover of authors: in 5 cases the e-mail address of the corresponding author did not exist anymore, possibly because authors had moved to different institutions or private practice. In further 4 papers, it was specified that the research was based on a (university or doctoral) dissertation thesis, which may imply (although not necessarily) discontinuation in the managing or supervising interventions after graduation/PhD has been achieved.

As for the strategy to visit websites, in at least 7 cases we achieved further information with respect to those included in the published paper. For example, in the website of Hadassah University Hospital, Jerusalem [23] it is specified that ‘*headache is treated with a multidimensional approach taking into account biological, emotional, social and functional components. Behavioral interventions are regularly offered to patients as complementary therapies. Patients can choose their preferred treatment and combine treatments as needed. Collaboration with the Medical Psychology Unit of the Hospital is available for patients interested in psychological counselling*’. In the online pages of the Departments of Neurology and Emergency Medicine, NYU, Langone Medical Center, New York City [62] several treatments are described as offered to people with headache/migraine including evidence-based relaxation techniques, BFB, progressive muscle relaxation, CBT and a brief description of each treatment is given (see Table 1 and Supplementary Table). Internet and other technological instruments (i.e. apps) represent useful and easy-to-access source of information for potential researchers and patients, that should be constantly updated to avoid becoming further barrier to the accessibility of interventions.

This paper presents both strengths and limitations.

As regards strengths, this systematic review focuses on an original topic not sufficient explored yet, which is stressing the existing gap between research and clinical practice and informing researchers, clinicians and health agencies on barriers and variables favoring implementation of evidence-based psychological approaches to CH in adult. Moreover, our search approach was made up of three complementary strategies (literature, direct contact with authors, websites search) allowing a more comprehensive understanding of the implementation of psychological interventions.

As regards limitations, it is not custom in scientific literature to provide information on what happens after the end of published research. Second, we limited literature research to the period 2008–2018 therefore considering only the more recent studies. Thirdly, we based our reports on what was described on papers or websites, but we cannot exclude that some papers did not specify i.e. the presence and nature of a grant in the acknowledgments section. Also, websites are not constantly updated, and the lack of information on a hospital/clinical center website does not necessarily mean that the interventions are not actually implemented. Finally, we should keep in mind that more recent published studies would have had less chance to be implemented in the last few months.

## Conclusions

Despite limitations, the present paper explored studies reporting evidence-based psychological interventions for CH, also including MoH, showing that they are feasible, effective and well-tolerated, alone or as part of multi-componential approaches. By contrast, very few studies provided information on the actual implementation of results into clinical routinely settings. The examination of barriers and factors affecting the accessibility and generalizability of interventions, together with analysis of cost-effectiveness, should be included in future studies. Moreover, including in the professional training of clinical scientist contents regarding dissemination and implementation of interventions can represent a valid way to strength the link between future research and clinical practice.

## Supplementary information

**Additional file 1.** Survey.

**Additional file 2: Supplementary Table.** Other variables extracted from the 28 selected studies (in chronological order).

## Data Availability

All used data are reported in Table 1 and Supplementary Table (Additional file 2). The survey sent to the corresponding authors by e-mail is available as Additional file 1.

## Abbreviations

ACT: Acceptance and Commitment Therapy
BFB: Biofeedback
CBT: Cognitive Behavioral Therapy
CH: Chronic Headache
ICCPN: Italian Consensus Conference on Pain in Neurorehabilitation
ICHD: International Classification of Headache Disorders
IRCCS: Institute for Treatment and Research
MIDAS: Migraine Disability Assessment
MoH: Medication overuse Headache
PRISMA: Preferred Reporting Items for Systematic Reviews and Meta-Analyses
QATSDD: Quality Assessment tool
RCT: Randomized Controlled Trial
TAU: Treatment As Usual
TTH: Tension Type Headache
