# Supplementary material for: From research to clinical practice: a systematic review of the implementation of psychological interventions for chronic headache in adults
Source: BMC Health Serv Res. 2020 May 25;20:459. doi: 10.1186/s12913-020-05172-y (PMC7247180; doi:10.1186/s12913-020-05172-y)
Supplement: Supplementary file 1 — Additional file 1. Survey. [file 12913_2020_5172_MOESM1_ESM.doc]

***A 5-minutes survey by University of Verona (Italy) about your paper on psychological intervention for migraine***

• Question 1. To date, has the intervention described in your published paper been implemented in clinical practice? (YES-NO)

• Question 2. Currently, is the intervention routinely implemented? (YES-NO)

• Question 3. The implementation of the intervention in the clinical practice required adaptations or adjustments? (YES-NO)

If YES, please list them here: _______________________

• Question 4. Which factors represented barriers or constituted an obstacle to the implementation of the intervention in the routine clinical practice? (more than one answer is possible)

a) The intervention did not result effective

b) Lack of funding

c) Lack of qualified staff to carry out the intervention

d) Lack of commitment and support by the management

e) Lack of facilities (e.g. an adequate room for group activities)

f) Reasons connected to patients: scarce compliance/low adherence

g) Reasons connected to patients: accessibility issues (i.e. distance from the hospital)

h) Reasons connected to patients: socio-economic factors (i.e. educational status, healthcare insurance etc)

i) Other (please specify): _______________________

Please write here the letter(s) of the answer(s) you chose: _______________________

• Question 5. According to you, which factors are more important for a successful implementation of the intervention researched in your study? (more than one answer is possible)

a) Availability of a Clinical Psychological Service or qualified psychological staff

b) Availability of a multidisciplinary equipe

c) Presence of commitment and support by the management

d) Resources (i.e. funding)

e) Presence of adequate facilities (e.g. an adequate room for group activities)

f) Patients Compliance

g) Other (please specify) :____________________

Please write here the letter(s) of the answer(s) you chose: _______________________

Thank you for your valuable help
