# Supplementary material for: From research to clinical practice: a systematic review of the implementation of psychological interventions for chronic headache in adults
Source: BMC Health Serv Res. 2020 May 25;20:459. doi: 10.1186/s12913-020-05172-y (PMC7247180; doi:10.1186/s12913-020-05172-y)
Supplement: Supplementary file 2 — Additional file 2: Supplementary Table. Other variables extracted from the 28 selected studies (in chronological order). [file 12913_2020_5172_MOESM2_ESM.doc]

| **paper** | **Hospital/clinical centre mentioned in the study (involved in recruitment and/or intervention)** | **website (last consultation September 27, 2019)** | **participants included in the study (analysed)** | **recruitment** | **individual/group (+additional delivery mode)** | **lenght of intervention** | **adherence to intervention** | **main results** | **funding** | **implementation into clinical context at the end of research (as specified in the paper and/or website)** |
| --- | --- | --- | --- | --- | --- | --- | --- | --- | --- | --- |
| D'Souza et al 2008 [52] | no | no | 51 TTH (50) 90 migraine (85) | undergraduate students | individual (audiotape)(+ homework) | 2 weeks | 96% | RT: ↑ calmness; ↓ headache frequency and disability (compared to WED and control group; only in TTH); ↓ pain severity (relative to control group; only in migraine). WED: ↑ negative mood | partly funded (private+public) | ns |
| Matchar et al 2008 [48] | 3 primary care settings sites | no | 614 (437) | referred by physician | individual+group | 6 months | 71% | ↓ MIDAS scores, depressive symptoms, worry about headache; ↑ quality of life, satisfaction in headache management in the intervention group compared to usual care group | funded *(but clinical visits excluded and paid by patients’ health insurance)* | ns |
| Sauro et al 2008 [11] | Alberta Health Service, Alberta | https://www.albertahealthservices.ca/ (CHAMP program:https://www.albertahealthservices.ca/services/page3672.aspx) | 139 (self-management module) | referred by physician | individual+group (+homework) | 5 weeks | 95% (self-management module) | The program was well accepted by many patients and perceived to be useful by them. Headache related disability as measured by standard measures was significantly reduced after participation in the program. | funded (private+public) *Fundingwas obtained for salaries for a nurse, an occupational therapist, and a half-time psychologist for three years* | Authors state that 'with appropriate support from funding agencies, a multidisciplinary headache program as CHAMP can be successfully established as part of the Canadian public healthcare system'. A specific section of the Alberta Health Service website is dedicated to the CHAMP program (with info on workshop schedule, power point slides, headache diary, links to local chronic pain programs) |
| Grazzi et al 2009 [67] | Neurological Institute “C. Besta” IRCCS Foundation, Headache and Neuroalgology Unit, Milan | https://www.istituto-besta.it/cefalee-dolore (only Italian version available) | 84 | patients treated for MoH by a day-hospital withdrawal | group (+homework) | 8 weeks | 93% (RT group) | RT: greater compliance compared to medication | ns | on last consultation date (September 27, 2019) the webpage focusing on treatment offered for chronic migraine was under construction |
| **Paper** | **Hospital/clinical centre mentioned in the study (involved in recruitment and/or intervention)** | **website (last consultation September 27, 2019)** | **participants included in the study (analysed)** | **recruitment** | **individual/group (+additional delivery mode)** | **lenght of intervention** | **adherence to intervention** | **main results** | **funding** | **implementation into clinical context at the end of research (as specified in the paper and/or website)** |
| Gunreben-Stempfle et al 2009 [12] | Pain Center, University of Erlangen-Nuremberg, Erlangen | http://www.schmerzzentrum.uk-erlangen.de/ (no English version available) | 42 (experimental group, compared to previously published control samples data) | referred by family physicians or specialists | individual+group (+homework) | 8 weeks | 100% | ↓ migraine and TTH headache days/month, frequency of migraine attacks, and depressive symptoms with greater effects of the 96 hours multidisciplinary program compared to standard care and to the low intensity program | *costs covered by patients’ health insurance* | ns |
| Holroyd et al 2009 [60] | no | no | 203 (169) | ns | individual (+telephone support) | 8 weeks | 83% | in the two SMT conditions: ↓ headache disability | funded (public) | ns |
| Fritsche et al 2010 [53] | seven German headache centers located all over Germany | no | 182 | general population; referred by medical practices | group (+homework) | 5 weeks | 95% (experimental group) | ↓ headache days in both MCP and bibliotherapy groups, with beneficial effect greater in patients with a high rather than a low headache frequency | funded (public) | ns |
| Gaul et al 2011 [13] | Headache Center, Department of Neurology, University Hospital Essen | https://www.uk-essen.de/index.php?id=2376&L=1 www.uni-essen.de/neurologie (no english version available) | 362 (295) | headache centre | individual+group | 5 days | 81% | ↓ headache frequency | *clinical costs covered by patients' health insurance* | In the hospital webpage is specified that the centre is specialized in the diagnosis and treatment of headaches with 4,500 headache patients every year (both out-and inpatients). A multidisciplinary team is involved in patients management (psychologists are not mentioned). No reference to MTP is available |
| Hedborg and Muhr 2011 [14] | no | no | 83 (76) | general population | individual (online) | 9 months | 91.5% | ↓ migraine frequency in the groups receiving multimodal behavioral treatment (with and without hand massage) compared to control group | funded (private+public) | ns |
| **Paper** | **Hospital/clinical centre mentioned in the study (involved in recruitment and/or intervention)** | **website (last consultation September 27, 2019)** | **participants included in the study (analysed)** | **recruitment** | **individual/group (+additional delivery mode)** | **lenght of intervention** | **adherence to intervention** | **main results** | **funding** | **implementation into clinical context at the end of research (as specified in the paper and/or website)** |
| Abdoli et al 2012 [54] | Nursing and Midwifery Care Research Centre, Nursing and Midwifery Faculty, Isfahan University of Medical Sciences, Isfahan | https://english.mui.ac.ir/ | 60 | general population; referred by physician | individual (audiotape in GI group) | 5 weeks | 100% | GI (both tape and perceived happy memory): ↓ headache intensity, frequency and duration compared to control group | funded (public).*Certified psychologist who administered intervention was part of the clinical staff* | ns |
| Bembalgi et al 2012 [65] | no | no | 121 (96) | patients from neurology clinic | individual | ns (15 sessions) | 79% | All the three forms of BF (auditory, visual and combined) are effective in treatment of TTH ( ↑ quality of life) with combined BF slightly more effective than isolated auditory or visual BF | ns | ns |
| Ezra et al 2012 [23] | Hadassah University Hospital, Jerusalem | http://www.hadassah-med.com/ (headache: http://www.hadassah-med.com/medical-care/departments/neurology/headaches.aspx) | 98 (92) | headache centre | individual | ? | 62% | HR: ↑ symptom relief and compliance compared to amitriptyline | none | In the paper is specified that patients were routinely given the option to choose (and cross-over) between 2 standard protocol: HR vs amitriptyline. HR was performed during standard length neurology clinic appointments. In the Hospital website pages is specified that headache is treated with a multidimensional approach taking into account biological, emotional, social and functional components. RT, BFB, HR, GI are regularly offered to patients as complementary therapies. Patients can choose their preferred treatment and combine treatments as needed. A collaboration with the Medical Psychology Unit of the Hospital is available for patients interested in psychological counseling. |
| **Paper** | **Hospital/clinical centre mentioned in the study (involved in recruitment and/or intervention)** | **website (last consultation September 27, 2019)** | **participants included in the study (analysed)** | **recruitment** | **individual/group (+additional delivery mode)** | **lenght of intervention** | **adherence to intervention** | **main results** | **funding** | **implementation into clinical context at the end of research (as specified in the paper and/or website)** |
| Mo'tamedi et al 2012 [61] | Baqiyatallah Hospital (Tehran) | https://baq.bmsu.ac.ir/portal/home/?283090/english (no information on headache treatment in the english version of the site) | 30 (26) | specialized headache clinic patients | group | 8 weeks | 86% | ACT: ↓ disability and affective distress (but not in reported sensory aspect of pain) compared to TAU | ns *(who delivered intervention was part of the hospital staff)* | ns |
| Ruehlman et al 2012 [50] | GOALISTICS, Arizona | https://pain.goalistics.com/ | 330 (305) | specialized pain sites | individual (online) | 6 weeks | 92% | ↓ pain severity, pain-related interference, emotional burden, perceived disability, catastrophizing, pain-induced fear, depression, anxiety, and stress. ↑ knowledge about the principles of chronic pain and its management | funded (public) | The CPMP is described in detail in the website and is available for purchase. It is hence routinely applied. |
| Wallasch et al 2012 [15] | Headache Center Berlin at the Sankt Gertrauden Krankenhaus Berlin | http://www.sankt-gertrauden.de/ (no English version available) | 201 | referred by general practitioners (primary care), neurologists, or insurance companies when headache treatment failed | individual+group (+hospitalization) | 5 days | 100% | ↓ headache frequency, headache-related disability, lost work/school time, anxiety and depression, amount and intake frequency of acute medication | ns *(intervention providers are part of the staff of the centre)* | ns |
| Slavin-Spenny et al 2013 [69] | no | no | 147 (127) | college students | group | 2 weeks | 86.4% | AAET: ↓ alexithymia, ↑ emotional processing and assertiveness; both AAET and RT: ↑ self-efficacy to manage headache and improved headache outcomes relative to controls | funded (private+public) | ns |
| Cathcart et al 2014 [55] | no | no | 58 (42) | general population | group (+homework) | 3 weeks | 72% | ↓ headache frequency, ↑ mindfulness score compared to waiting list group | ns | ns |
| **Paper** | **Hospital/clinical centre mentioned in the study (involved in recruitment and/or intervention)** | **website (last consultation September 27, 2019)** | **participants included in the study (analysed)** | **recruitment** | **individual/group (+additional delivery mode)** | **lenght of intervention** | **adherence to intervention** | **main results** | **funding** | **implementation into clinical context at the end of research (as specified in the paper and/or website)** |
| Day et al 2014 [63] | Kilgo Headache Clinic (Northport, Alabama); University of Alabama Psychology Clinic (Tuscaloosa, Alabama) | https://psychologyclinic.ua.edu/ (no information on headache treatment) | 36 | general population+referred by physician | group (+homework) | 8 weeks | 66% | MBCT: ↑ self-efficacy and pain acceptance; ↓ pain interference and pain catastrophizing (compared to DT) | funded (not specified) | ns |
| Martin et al 2014 [66] | no | no | 127 (67) | general practice referrals; general population | individual | 8 weeks | 53% | LCT: greater improvement on all measures of headaches and medication consumption compared to the other three conditions | funded (public) | ns |
| Christiansen et al 2015 [56] | Department of Psychology, Outpatient Clinic of Behavior Therapy, University of Hamburg; University Medical Center Hamburg-Eppendorf, Hamburg, Hamburg | https://www.uke.de/english/ | 87 (80) | patients of the Outpatient Clinic of Behavior Therapy | individual+group (+homework) | 10 weeks | 92% | ↓ headache intensity, headache frequency and catastrophizing; ↑ Coping strategies | none | ns |
| Cousins et al 2015 [68] | no | no | 73 (56) | referred by neurologists and family doctors | individual (+homework and email/telephone support) | 5 weeks | 76.7% | a future definitive trial treatment is feasible, with small modifications of protocol, within a UK National Health system context. | partly funded (private+public)*The treatment (therapist) was funded by a charity* | In the paper is specified that 'this is pilot study to provide design information necessary for a future definitive trial of the treatment within a UK National Health system context' |
| **Paper** | **Hospital/clinical centre mentioned in the study (involved in recruitment and/or intervention)** | **website (last consultation September 27, 2019)** | **participants included in the study (analysed)** | **recruitment** | **individual/group (+additional delivery mode)** | **lenght of intervention** | **adherence to intervention** | **main results** | **funding** | **implementation into clinical context at the end of research (as specified in the paper and/or website)** |
| Bakhshani et al 2016 [64] | Hospitals affiliated to Zahedan University of Medical Sciences (Zahedan) | http://en.zaums.ac.ir/ (no information on headache treatment on the 3 English version websites of the affiliated hospitals) | 40 | referred by  neurologist/psychiatrist | group (+homework) | 8 weeks | 92.5% | ↓ pain, ↑ quality of life in the experimental group compared to the 'drug only' group | partly funded | ns |
| Rausa et al 2016 [51] | Headache Center of IRCCS Institute of Neurological Sciences (Bologna) | https://www.ausl.bologna.it/isnb (only Italian version available) | 72 (27) | specialized headache clinic patients | individual | 9 weeks | 37.5% | ↓ headache frequency, amount of drug intake; ↑ active coping with pain and greater number of patients that returned episodic in BF compared to control group | funded (private) | It is specified in the website that diagnostic and therapeutic approaches are offered to patients with primary acute migraine. BFB is not mentioned |
| Smitherman et al 2016 [57] | no | no | 32 (31) | neurology clinic; university | group | 6 weeks | 97% | CBT: ↓ headache frequency and improvement in sleep parameters | funded (private) | ns |
| Grazzi et al 2017 [59] | Neurological Institute “C. Besta” IRCCS Foundation, Headache and Neuroalgology Unit, Milan | https://www.istituto-besta.it/cefalee-dolore (only Italian version available) | 44 | headache centre | group (+homework) | 6 weeks | 100% | both MBSR and medication: ↓ headache frequency, use of medication, MIDAS score, BDI | funded (private+public) | on last consultation date (September 27, 2019) the webpage focusing on treatment offered for chronic migraine was under construction |
| **Paper** | **Hospital/clinical centre mentioned in the study (involved in recruitment and/or intervention)** | **website (last consultation September 27, 2019)** | **participants included in the study (analysed)** | **recruitment** | **individual/group (+additional delivery mode)** | **lenght of intervention** | **adherence to intervention** | **main results** | **funding** | **implementation into clinical context at the end of research (as specified in the paper and/or website)** |
| Krause et al 2017 [49] | Cleveland Clinic Foundation | https://my.clevelandclinic.org/ (headache: https://my.clevelandclinic.org/health/articles/8262-headache-treatment-overview; https://my.clevelandclinic.org/health/articles/11664-headache-management-relaxation-and-other-alternative-approaches) | 379 (348) | patients who have received prior medical care for headaches either at their clinic or elsewhere | individual+group | 3 weeks | 91,80% | ↓anxiety, depression and reactivity to stress; ↓ headache pain and functional impairment | none | A detailed description of headache diagnostic phase and therapeutic approaches is given in the website including education, stress management, biofeedback, relaxation techniques, CBT. It is not clear whether the described treatments are all available at the Clinic |
| Wachholtz et al 2017 [58] | no | no | 92 (83) | undergraduate students, general population | individual (+homework) | 4 weeks | 90% | spiritual meditation: ↓ migraine frequency, medication intake compared to the other groups | funded (public) | ns |
| Minen et al 2019 [62] | Departments of Neurology and Emergency Medicine, NYU, Langone Medical Center, New York City | https://nyulangone.org/ (migraine:https://nyulangone.org/conditions/migraine-in-adults; headache: https://nyulangone.org/conditions/headache-in-adults) | 12 | Departments of Neurology and Emergency Medicine | individual (app) | ns | 100% | RELAXaHEAD app was acceptable and useful for migraine participants; PMR delivered through RELAXaHEAD improved stress and mood | funded (private+public) | In the Medical Centre website is specified that several treatments are offered to people with headache/migraine including evidence-based relaxation techniques, BFB, progressive muscle relaxation, CBT. A brief description of each treatment is given. RELAXaHEAD app is not mentioned but in the paper is specified that future large trial will be implemented to test its feasibility for people with migraine |

**Supplementary Table.** Other variables extracted from the 28 selected studies (in chronological order)

AAET= Anger Awareness and Expression Training; ACT= Acceptance and Commitment Therapy; BDI=Beck Depression Inventory; BFB=biofeedback; CBT= Cognitive Behavioral Therapy; CHAMP= Calgary Headache Assessment And Management Program; CPMP= Chronic Pain Management Program; DT= delayed treatment; GI= guided imagery; LCT= Learning to Cope with Triggers; HR=hypnotic relaxation; MBCT= Mindfulness-Based Cognitive Therapy; MBSR= Mindfulness-Based Stress Reduction training; MIDAS=Migraine Disability Assessment scale; MoH= Medication overuse Headache; MTP= Multidisciplinary Treatment Program; ns= not specified; PMR= Progressive Muscle Relaxation; RCT=Randomized Controlled Trial; RT= Relaxation Training; SMT= Stress-Management Therapy; TAU= Treatment As Usual; TTH= Tension Type Headache; WED: Written Emotional Disclosure
